# Beta-amyloid peptides(1–42) and (1–40) in the cerebrospinal fluid during pregnancy: a prospective observational study

**DOI:** 10.1007/s00737-020-01072-6

**Published:** 2020-10-02

**Authors:** Cristina Alomar-Dominguez, L. Dostal, J. Thaler, G. Putz, C. Humpel, W. Lederer

**Affiliations:** 1 Department of Anesthesiology and Critical Care Medicine, Medical University of Innsbruck, Anichstrasse 35, 6020 Innsbruck, Austria; 2 Department of Medical Statistics, Informatics and Health Economics, Medical University of Innsbruck, Innsbruck, Austria; 3 Psychiatric Laboratory, Department of Psychiatry, Psychotherapy and Psychosomatics, Medical University of Innsbruck, Innsbruck, Austria

**Keywords:** Beta-amyloid, Biomarkers, Growth factors, Cerebrospinal fluid, Pregnancy

## Abstract

To evaluate changes in concentrations of selected biomarkers, neurotrophic factors, and growth factors in the cerebrospinal fluid during pregnancy. A prospective observational study was conducted in 32 pregnant women undergoing gynecological and obstetrical surgery under spinal anesthesia in a university hospital. Beta-amyloid(1–42) and beta-amyloid(1–40) peptides, brain-derived neurotrophic factor, glial cell line-derived neurotrophic factor, and vascular endothelial growth factor were analyzed in cerebrospinal fluid using an enzyme-linked immunosorbent assay. Eight women in second trimester pregnancy who underwent spinal anesthesia for gynecological or obstetrical surgery were compared with 24 matched women in third trimester pregnancies. CSF concentrations of beta-amyloid(1–42) were significantly higher in third trimester pregnancies (*p* = 0.025). During third trimester, the beta-amyloid ratio correlated with the vascular endothelial growth factor (*r*_s_ = 0.657; *p* = 0.008). Higher concentrations of beta-amyloid(1–42) in cerebrospinal fluid of third trimester pregnancies and correlations between the beta-amyloid ratio and the vascular endothelial growth factor support the hypothesis that beta-amyloid peptides are involved in complex adaptive brain alterations during pregnancy.

## Introduction

Pregnancy is associated with profound physiological changes, including long-lasting alterations in the maternal brain such as neurogenesis and synaptic plasticity (Hoekzema et al. 2017). Peripartal remodeling of the brain substance is of vital importance, not only for proper development of the fetus but also for the mother’s mental health (Hillerer et al. 2014).

Several biomarkers and neurotrophic factors have been reported to take part in the physiological brain changes occurring during pregnancy. For instance, increased serum levels of vascular endothelial growth factor (VEGF) during pregnancy enhance blood-brain barrier permeability (Schreurs et al. 2012). Duman and Monteggia (2006) found that decreased levels of brain-derived neurotrophic factor (BDNF) can contribute to neuronal atrophy of certain limbic structures. In addition, Garcés et al. (2014) reported that serum levels of BDNF decrease during pregnancy and considered BDNF as a regulator of several metabolic functions during pregnancy. Glial cell line-derived neurotrophic factor (GDNF) is a proliferation factor that contributes to the development of malignant brain gliomas in the rat brain (Wiesenhofer et al. 2000). It is not clear whether GDNF plays a role during pregnancy.

Amyloid precursor protein (APP), a trans-membranous protein most commonly found in brain and platelets, regulates synapse formation and neural plasticity and induces axogenesis and neurite growth (Priller et al. 2006; Halle et al. 2008). APP is sequentially cleaved by membrane-bound secretases to generate different beta-amyloid peptides, of which beta-amyloid(1–40) is the most abundant (Priller et al. 2006; Halle et al. 2008). Beta-amyloid peptides are products of normal cell metabolism (Canobbio et al. 2015) and have trophic properties: for example, they capture redox metal ions and thus dampen oxidative stress (Atwood et al. 2003). In a recent study (Lederer et al. 2020), we reported that beta-amyloid peptides correlate with placental dysfunction.

In recent years, intensive research conducted in rodents has focused on underlying mechanisms of brain alterations during pregnancy (Barba-Müller et al. 2018). Research involving the human brain was performed mostly through neuroimaging techniques such as magnetic resonance (Barba-Müller et al. 2018). Only a few studies have analyzed cerebrospinal fluid (CSF) of pregnant women, and in most of them, CSF samples were taken before delivery by cesarean section (Ciampa et al. 2018; Altemus et al. 2004). Analysis of CSF in early pregnancy could provide key information on normal physiological pathways of maternal brain adaptation.

In the present study, we analyzed beta-amyloid(1–42), beta-amyloid(1–40), BDNF, GDNF, and VEGF in CSF of women during second trimester of pregnancy and before delivery by cesarean section in order to detect variations in concentration during healthy pregnancies.

## Materials and methods

### Study background

The purpose of the study was to evaluate changes in concentrations of selected biomarkers and neurotrophic factors in CSF during pregnancy. As access to CSF is limited to medical indications, only pregnant women undergoing surgical operations in spinal anesthesia were subjects of this investigation.

### Study design

The study was conducted in accordance with the Declaration of Helsinki (World Medical Association 2013) and was approved by the Ethics Committee of the Medical University of XXX (blinded) (AN2015-0156 351/4.4). Women admitted to the Department of Gynecology and Obstetrics of XXX(blinded) Medical University Hospital, who underwent spinal anesthesia during pregnancy, were consecutively enrolled. Written informed consent was obligatory for participation. Data were collected from August 2015 to June 2018 mainly during normal working hours.

### Study population

Inclusion criteria were pregnant women > 18 years in good general health undergoing gynecological or obstetrical operations in spinal anesthesia.

Exclusion criteria were pregnant women < 18 years, chronic renal and hepatic disease, major neurological disease, past or present major psychiatric disorders, hypertensive disorders of pregnancy including gestational hypertension and preeclampsia, HELLP syndrome, low platelet count < 100,000/μL as relative contraindication against spinal anesthesia, intrauterine growth restriction, anomalies of the placenta in sonography, and lack of consent of the participants.

### Data collection

Medical history and chronic medications other than multivitamins, magnesium, and iron supplementation were recorded. Blood chemistry (hemoglobin, blood sugar, and protein levels), liver, and kidney function parameters (blood urea nitrogen, glomerular filtration rate, creatinine, and proteinuria) were obtained from patients’ records. Systolic and diastolic blood pressure, pulse rate, and oxygen saturation were measured non-invasively prior to spinal anesthesia. Demographics, clinical characteristics, and laboratory findings of the study participants were recorded on a working chart. Gestational age at the time of the procedure was specified as second trimester of pregnancy from week 13 to the end of week 28 and as third trimester of pregnancy from week 29 to delivery (US Department of Health and Human Services 2019).

### Sampling and analytical techniques

Spinal block was performed according to standard procedures of the Department of Anesthesiology and Critical Care Medicine. After puncture of the subarachnoid space and immediately before injection of the anesthetic, 1 mL of CSF was collected in a sterile polypropylene tube (Lederer et al. 2016). The samples were immediately transported to the Psychiatric Laboratory of the Department of Psychiatry and stored at − 80 °C prior to processing (Schooneboom et al. 2005). For analysis all CSF samples were thawed, and concentrations of beta-amyloid(1–42), beta-amyloid(1–40), (Assay Innotest®, Fujirebio Europe, B-9052 Gent, Belgium), brain-derived neurotrophic factor (BDNF), glial cell line-derived neurotrophic factor (GDNF), and vascular endothelial growth factor (VEGF) (Promega Emax® ImmunoAssay (Promega GmbH, Mannheim, Germany) were measured by enzyme-linked immunosorbent assay (ELISA) according to the manufacturer’s instructions.

### Statistical analysis

Surgical operations in spinal anesthesia during second trimester of pregnancy are rare. As the number of women in the second trimester of pregnancy in our study is low, they were matched with women in the third trimester pregnancy in a ratio of 1:3 according to age. Continuous variables were reported as mean ± standard deviation (SD) if there was normal distribution and as median and range if there was no normal distribution. The differences in the outcome characteristics between women in second trimester pregnancy and the average of their three matched controls (women in third trimester pregnancy) were compared using the paired *T* test in biomarkers for normally distributed outcomes and using the Wilcoxon test for not normally distributed outcomes. Associations between CSF levels of biomarkers and growth factors were examined using Pearson’s correlation coefficient in the case of normal distribution, Spearman’s correlation coefficient otherwise. Calculations were conducted with SPSS 21.0 (IBM SPSS Statistics Standard). Results were significant at the 0.05 level (2-tailed).

## Results

### Patient characteristics

**Fig. 1:**
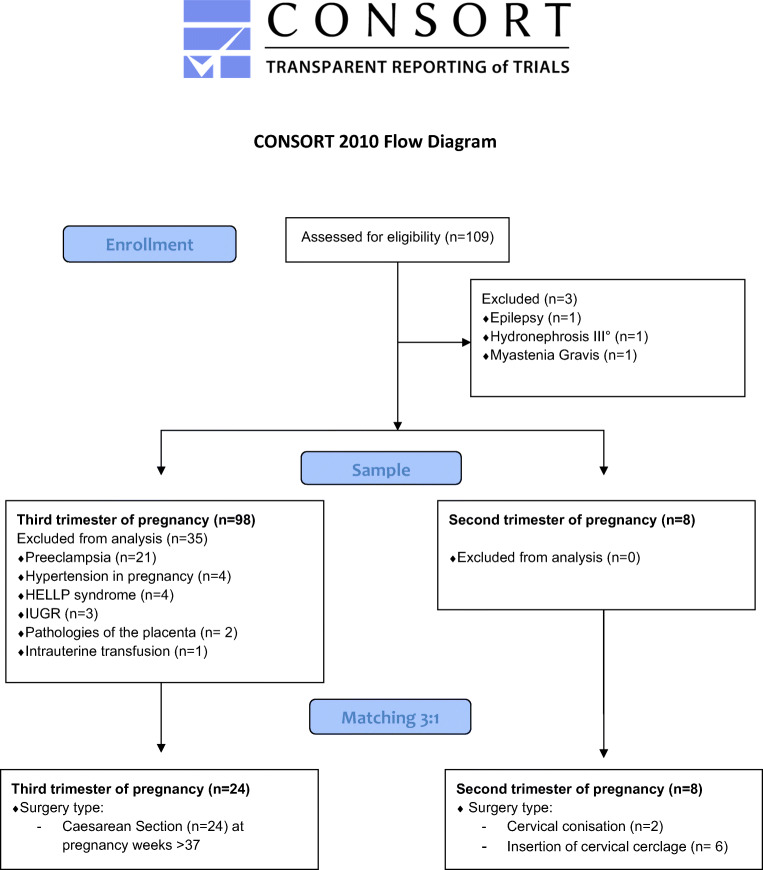
Flow diagram of the study population (CONSORT 2010)

**Fig. 2:**
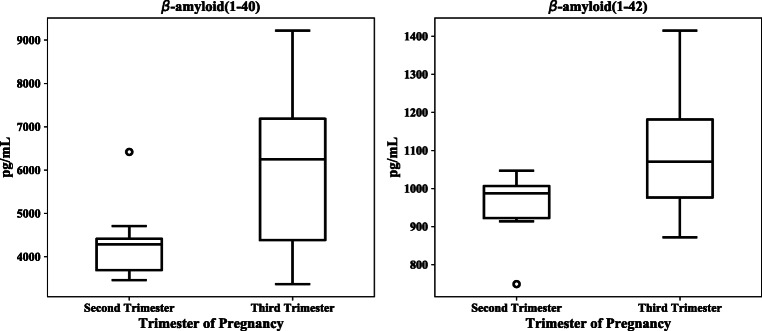
Concentrations of beta-amyloid(1–42) and (1–40) peptides in the cerebrospinal fluid (CSF) of 32 pregnant women according to the trimester of pregnancy (Box plot)

Eight women in second trimester of pregnancy who underwent spinal anesthesia for gynecological or obstetrical surgery were consecutively enrolled. Patients were treated for cervical incompetence (*n* = 6) or for dysplastic lesions of the uterine cervix (*n* = 2). Sixty-three healthy pregnant women in third trimester of pregnancy (weeks 29 to 41) underwent cesarean section in spinal anesthesia. For comparison of both groups, cases from second and third trimester of pregnancy were matched in a ratio of 1:3 according to age (Fig. 1). Twenty-four matched women formed the control group (Consort flow diagram, Fig. 2). Medical indications for cesarean section in the control group included previous cesarean section (*n* = 13), anomalies of the fetal position (*n* = 9), placenta previa (*n* = 1), and perineal tear in previous vaginal births (*n* = 1). Four women in third trimester pregnancies diagnosed and appropriately treated for gestational diabetes presented normal blood sugar levels at time of delivery.

Demographic data are displayed in Table 1. Age, height, and weight did not differ statistically among the groups. Findings regarding cardiorespiratory function on day of spinal anesthesia were similar in both groups. Blood analysis was similar among the groups except for fibrinogen (*p* = 0.005), which was significantly higher in third trimester pregnancies (Table 1).

**Table 1:** Demographic data, blood analysis with chemistry, liver function, clotting system, kidney function, and urinalysis in eight women in the second trimester of pregnancy and 24 women in the third trimester of pregnancy matched in age 1:3

	Second trimester of pregnancy (weeks 14–22) *n = 8*	Third trimester of pregnancy (week 37–41) *n = 24*	*p* value
Demographic data	Mean ± SD Median (IQR)	Mean ± SD Median (IQR)	
Age (years)	34.0 ± 5.8	33.8 ± 5.5	0.104
Height (cm)	168.8 ± 8.2	164.9 ± 7.8	0.277
Weight^1^ (Kg)	66.9 ± 11.1	64.4 ± 12.7	0.876
BMI^1^ (Kg/m^2^)	23.9 ± 3.1	23.8 ± 5.1	0.670
Gravidities	3.8 ± 1.8	2.5 ± 1.4	0.155
Deliveries	0.8 ± 0.8	0.7 ± 0.9	0.798
Aborts	2.0 ± 1.5	0.8 ± 1.4	0.134
Cardiorespiratory function			
Systolic blood pressure (mmHg)	124.6 ± 12.1	122.7 ± 9.3	0.585
Diastolic blood pressure (mmHg)	80.3 ± 7.1	78.7 ± 1.9	0.561
Heart rate (1/min)	84.0 ± 11.7	89.1 ± 14.9	0.299
Oxygen saturation (%)	97.8 ± 0.9	97.6 ± 1.6	0.802
Blood analysis			
Hemoglobin (mg/dL)	11.9 ± 1.1	12.1 ± 1.2	0.670
Glucose (mg/dL)	84.2 ± 8.1	90.9 ± 19.9	0.186
Platelets (10^3^/μL)	212.6 ± 33.4	237.8 ± 62.7	0.144
Protein (mg/dL)	6.6 ± 0.8	6.4 ± 0.8	0.717
AST (GOT) (mg/dL)	15.6 ± 3.3	19.0 ± 4.6	0.037
Lactate dehydrogenase (mg/dL)	157.8 ± 19.9	201.2 ± 50.7	0.102
Fibrinogen (mg/dL)	375.9 ± 50.6	438.5 ± 56.7	*0.027*
Prothrombin time (%)	103.1 ± 9.8	109.1 ± 9.1	0.249
Partial thromboplastin time (s)	27.0 ± 2.67	26.13 ± 2.0	0.374
Urea (mg/dL)	18.9 ± 4.0	16.0 ± 5.2	0.126
Creatinine (mg/dL)	0.6 ± 0.1	0.6 ± 0.1	0.091

Numerical data were expressed as mean and standard deviation and were analyzed with the paired *t* test in normally distributed biomarkers. Numerical data were expressed as median and interquartile range (IQR) and were analyzed with the Wilcoxon test for not normally distributed outcomes. Nominal data were expressed as frequencies and were analyzed with the chi-square test/Fisher’s exact test

1 at the beginning of the pregnancy

^a^BMI gain is the increase in BMI from onset of pregnancy to current BMI

Significant values are indicated in italics

### Biomarkers and neurotrophic factors

CSF concentrations of beta-amyloid(1–42) were significantly lower in second trimester pregnancies than at delivery (Table 2, Fig. 1). Beta-amyloid(1–40) concentrations were lower in trend, but were not significant (*p* = 0.069) (Table 2, Fig. 2). The beta-amyloid ratio decreased from second to third trimester in trend, but was not significant (*p* = 0.123) (Table 2). During the third trimester, the beta-amyloid ratio correlated with the vascular endothelial growth factor (*r*_s_ = 0.657; *p* = 0.008). When calculating ROC (receiver operating characteristics) with the area under the ROC curve (AUC, 0.719), there was an approximately 72% (95% CI: 0.453–0.984; *p =* 0.141) chance of distinguishing between the beta-amyloid ratio Aβ42/40 and CSF concentrations of beta-amyloid(1–42).

**Table 2:** Cerebrospinal levels of beta-amyloid(1–42), beta-amyloid(1–40), brain-derived neurotropic factor (BDNF), glial cell line-derived neurotrophic factor (GDNF), vascular endothelial growth factor (VEGF) in eight women in the second trimester of pregnancy, and 24 women in the third trimester of pregnancy matched in age 1:3

	Second trimester of pregnancy (weeks 14–28) *n = 8*	Third trimester of pregnancy (week 29–41) *n = 24*	*p* value
CSF biomarkers	Mean ± SD Median (IQR)	Mean ± SD Median (IQR)	
ß-Amyloid 1–42 (pg/mL)	955.0 ± 95.6	1125.8 ± 167.7	*0.025*
ß-Amyloid 1–40 (pg/mL)	4290.9 (3625–4515)	6250.0 (4330–7200)	0.069
ß-Amyloid ratio(1–42/1–40)	0.22 (0.21–0.25)	0.18 (0.16–0.24)	0.123
BDNF (pg/mL)	6.4 (4.3–6.7)	4.6 (3.1–12.0)	0.310
VEGF (pg/mL)	5.3 ± 3.1	5.8 ± 4.0	0.769
GDNF (pg/mL)	0.39 (0.16–0.47)	0.36 (0.24–0.55)	0.866

Numerical data were expressed as mean and standard deviation and were analyzed with the paired *t* test in normally distributed biomarkers. Numerical data were expressed as median and interquartile range (IQR) and were analyzed with the Wilcoxon test for not normally distributed outcomes

Significant values are indicated in italics

CSF concentrations of growth factors BDNF (*p* = 0.473), VEGF (*p* = 0.769), and GDNF (*p* = 0.635) did not differ among the groups. Concentrations of GDNF (*r*_s_ = 0.559; *p* = 0.030) correlated positively with BDNF.

## Discussion

We quantified CSF concentrations of beta-amyloid peptides and selected neurotrophic factors and growth factors in the second and third trimester of healthy pregnancies.

### Beta-amyloid peptides

In our study, concentrations of beta-amyloid(1–42) in CSF were found to be significantly higher in the third trimester than in the second trimester of pregnancy. The beta-amyloid(1–42/1–40) ratio was reported to be superior to CSF concentrations of beta-amyloid(1–42) and beta-amyloid(1–40) alone in early diagnosis of Alzheimer’s disease (Lewczuk et al. 2015). However, the decrease in the beta-amyloid ratio from second to third trimester was not significant in our study.

We attribute increasing beta-amyloid(1–42) concentrations to ongoing physiological changes, both structural and metabolic, in the maternal brain. Structural changes in the pregnant brain include neurogenesis, synaptic plasticity, and dendritic remodeling (Hillerer et al. 2014). Beta-amyloid(1–42) can directly activate microglia (Halle et al. 2008), a process known to accompany brain tissue remodeling (Ransohoff and Brown 2012). Metabolic changes during pregnancy such as increased insulin secretion (Barbour et al. 2007) and insulin resistance occur physiologically in order to support the demands of the fetus (Wilcox 2018). Insulin was reported to reduce beta-amyloid cleavage (Farris et al. 2003) and to increase CSF beta-amyloid concentrations in healthy older adults (Watson et al. 2003). Furthermore, hyperinsulinemia has been associated with a higher risk of developing Alzheimer’s disease (Luchsinger et al. 2004), a protein aggregation disease in which beta-amyloid accumulates and aggregates in the brain parenchyma and vessels (Canobbio et al. 2015). In our study, women with gestational diabetes (indicating increased insulin resistance) were treated appropriately and had normal blood sugar values. Beta-amyloid peptides in CSF did not correlate with blood sugar levels. Nevertheless, physiologically increased insulin levels during pregnancy may cause a rise in CSF beta-amyloid peptides. We propose that higher beta-amyloid concentrations in third trimester pregnancies are a consequence of physiologically upregulated metabolism during healthy pregnancy.

Beta-amyloid(1–40) is the most abundant species, whereas beta-amyloid(1–42) accounts for only 2–5% (Canobbio et al. 2015) of all beta-amyloid proteins. Because of its fibrillogenic properties, beta-amyloid(1–42) can misfold, aggregate, and accumulate in the brain parenchyma, typical of protein aggregation and conformational diseases (Labbadia and Morimoto 2015) such as Alzheimer’s disease. Recent findings indicate that a pathological metabolism of beta-amyloid peptides is involved in the pathogenesis of preeclampsia (Cheng et al. 2016), a severe pregnancy-related syndrome associated with a high mortality of mothers and children (Duley 2009). Buhimschi et al. (2014) found the presence of pathological beta-amyloid aggregates in placentas of women with preeclampsia, and Cheng et al. (2016) suggested preeclampsia could be a disease of protein misfolding. So far, we do not know whether beta-amyloid aggregates in the placental tissue of preeclamptic women correlate with beta-amyloid concentrations in cerebral tissue or CSF.

We here propose that beta-amyloid metabolism is upregulated during normal pregnancy. The question then arises whether a pathologic production or clearance defect of beta-amyloid peptides during pregnancy could be responsible for pregnancy-related central nervous system pathologies such as eclampsia, a severe complication of preeclampsia in which pregnant women suffer seizures (Duley 2009). In our previous work, beta-amyloid peptide levels in CSF were found to be lower in severe preeclampsia (Lederer et al. 2020). In a postmortem study, a lower beta-amyloid load in CSF of patients with Alzheimer’s disease correlated with increased beta-amyloid aggregates in brain parenchyma (Blennow and Zetterberg 2018). Interestingly, Alcantara-Gonzalez et al. (2019) found that injection of beta-amyloid could induce seizures in the rat brain. Whether an excess of beta-amyloid peptides in brain parenchyma due to a pathologic production or a clearance defect is causative for seizures in eclampsia is hypothetical and remains to be investigated.

### Neurotrophic factors and growth factors

Some neurotrophic factors and growth factors have been reported to influence the development of the materno-feto-placental unit (Schreurs et al. 2012). In this study, CSF concentrations of VEGF, BDNF, and GDNF did not differ between women in second trimester and women in third trimester of pregnancy. This corresponds with findings published by Garcés et al. (2014) regarding BDNF serum concentrations. In future studies, emphasis should be placed on evaluation of neurobehavioral development in offspring of women who suffered from preeclampsia.

## Limitations

First and foremost, the number of enrolled pregnancies in second trimester is small, as surgeries performed routinely during second trimester are rare. We are aware that a potential selection bias stems from the fact that only women who underwent surgical or obstetrical operations in spinal anesthesia could be enrolled. Furthermore, a distribution bias of sampling cannot be excluded as sampling of patient blood and CSF was performed mostly during the daytime, while diurnal variations in biomarkers cannot be excluded. In addition, duration of storage of samples varied among women enrolled and might have biased measurement results. Although ELISA assays for beta-amyloid were given high precision ratings, absolute beta-amyloid concentrations may vary between manufacturers (Van Waalwijk van Doorn et al. 2017).

## Conclusions

Increasing concentrations of beta-amyloid(1–42) in CSF of third trimester pregnancies support the hypothesis that beta-amyloid peptides are involved in the complex adaptive brain alterations during healthy pregnancy.
